# Commencement in the Buffalo Medical College—Names of the Graduating Class

**Published:** 1870-02

**Authors:** 


### Commencement in the Buffalo Medical College—Names of the Graduating Class.

The commencement exercises of the Buffalo Medical College were held February 22d, and were listened to by a large number of the friends of the Graduates and ol the institution.

The address to the Graduating Class by Rev. WALTER CLARK, D. D., was the chief feature of the occasion, and was listened to with much pleasure, not only by the profession, but by the entire audience. A vote of thanks was offered for the "interesting and instructive address, and a copy asked for publication."—This request having been granted, the public press seized it as a prize, and have used it so freely that we have little need to re-publish it for the benefit of our readers. It was able, scholarly, suggestive, and appropriate, doing honor both to the author and the occasion, and richly deserving the compliments it received.

By recommendation of the Faculty and Curators, the Council conferred the Degree of *Doctor in Medicine* upon the following gentlemen.

| | |
|---|---|
| Eugene Forman, | Sterling, Cayuga Co. N. Y. |
| Charles Berry, | Bell Plaine, Scott Co., Minn. |
| James Alexander Brush, | Sheakleyville, Mercer Co., Pa. |
| Erastus B. Letson, | Aurora, Erie Co N. Y. |
| Charles Benjamin Kibler, | Corry, Erie Co., Pa, |
| David H. McCluskey, | Pulaski, Lawrence Co., Pa. |
| Stewart Henry Benton, | Albion, Erie Co., Pa. |
| Robert Andrew Snodgrass, | Jamestown, Mercer Co., Pa. |
| Hugh Addison Davenny, | Youngsville, Warren Co., Pa. |
| John Deans Scouller, | Sparta, Randolph Co., Ill. |
| James Bignel Knapp, | Odessa, Schuyler Co., N. Y. |
| Charles Smith Pugsley, | Clarence Centre, Erie Co., N. Y. |
| Edward Elias Fuller, | Buffalo, Erie Co., N. Y. |
| George Rex Hinman, | Middleport, Niagara Co , N. Y. |
| Mathew J. McElhaney, | Hartstown, Crawford Co., Pa. |
| Robert Young Charles, | Angelica, Allegany Co., N. Y. |
| George Edward Blackham, | Dunkirk, Chautauqua Co., N. Y. |
| Walter Harvey Bills, | Warsaw, Wyoming Co., N. Y |
| John Boyes, | Orange, Schuyler Co., N. Y. |
| William Hartwell Chaddock, | Pewamo, Ionia Co., Mich. |
| Benjamin Franklin Leet, | Alliance, Stark Co., Ohio. |
| William Miles Wallis, | Darien, Genesee Co., N. Y. |
| David J. Wilson, | Westfield, Chautauqua Co., N. Y. |
| Dewitt C Boone, | North Cohocton, Steuben Co., N. Y. |
| James Sloan, | Buffalo, Erie Co., N. Y. |
| Charles Meine, | Germania, Potter Co., Pa. |
| Lucius William Byam, | Pavilion, Genesee Co., N. Y. |
| William Quincy Huggins, | Nunda, Livingston Co., N. Y. |
| John Morris Duff, | Holly, Orleans Co., N. Y. |
| Joseph Warren Hancock, B. S., | Red Wing, Goodhue Co., Min. |
| George Graves, | Herkimer, Herkimer Co., N. Y. |
| Otto Thoma, | Buffalo, Erie Co., N. Y. |
| George Addison Wallace, | Rochester, Munroe Co., N. Y. |
| James Kirke Stockwell, | East Wilson, Niagara Co., N. Y. |
| George Goodrich Carrol, A. M., | Rochester, Munroe Co., N. Y. |

| | |
|---|---|
| William Henry Allen, A. M., | Rochester, Munroe Co., N. Y. |
| Luther Hart Kitchel, A. B., | Middlebury, Addison Co., Vt. |
| Nelson Wilbur, A. B., | Orange, Schuyler Co., N. Y. |
| John Cynda Butterfield, | Churchville, Munroe Co., N. Y. |
| Richard Morris, | Delaware, Ontario. |
| William John Cronyn, | Lockport, Niagara Co., N. Y. |

The following Theses were found by the Faculty and Curators Worthy of *Honorable Mention.*

*Aphasia*, by Luther Hart Kitchell, A. B., which on account of its originality and accuracy of observation was also recommended for publication.

*Morbid Conditions and Chemical Analysis of the Uine*, by James Kirke Stockwell.

Thus closed, in some respects at least, the most successful Lecture term of the institution, which now appears as yearly increasing in all the essentials of true p osperity.

